# Avian Influenza Virus A(H5Nx) and Prepandemic Candidate Vaccines: State of the Art

**DOI:** 10.3390/ijms25158550

**Published:** 2024-08-05

**Authors:** Daniele Focosi, Fabrizio Maggi

**Affiliations:** 1 North-Western Tuscany Blood Bank, Pisa University Hospital, 56100 Pisa, Italy; 2 Laboratory of Virology, National Institute for Infectious Diseases “Lazzaro Spallanzani”-IRCCS, 00149 Rome, Italy; fabrizio.maggi@inmi.it

**Keywords:** avian influenza, influenzavirus, influenza virus, H_5_N_1_, clade 2.3.4.4b, genotype B3.13, live attenuated influenza, inactivated influenza vaccines

## Abstract

Avian influenza virus has been long considered the main threat for a future pandemic. Among the possible avian influenza virus subtypes, A(H_5_N_1_) clade 2.3.4.4b is becoming enzootic in mammals, representing an alarming step towards a pandemic. In particular, genotype B3.13 has recently caused an outbreak in US dairy cattle. Since pandemic preparedness is largely based on the availability of prepandemic candidate vaccine viruses, in this review we will summarize the current status of the enzootics, and challenges for H_5_ vaccine manufacturing and delivery.

## 1. A Brief History of Influenza Virus A(H_5_N_1_) 1996–2021

Human influenza viruses are currently categorized into types A, B, C, and D. While influenza A and B cause seasonal influenza epidemics (and A occasionally also pandemics), C causes mild illness in humans, and D affects primarily cattle but not humans. Human influenza A viruses preferentially bind sialic acid (SA)-α2,6 (human receptor), whereas avian influenza A viruses have a preference for α2,3 (avian receptor). The avian receptor can be further classified into two subtypes: influenza A viruses from chickens tend to prefer to bind to SA-α2,3-Gal-β1,4 (“chicken receptor”), whereas influenza A viruses from duck prefer SA-α2,3-Gal-β1,3 (“duck receptor”). For veterinary purposes, human influenza viruses are generally classified as either low-pathogenicity (LPAI) or high-pathogenicity avian influenza viruses (HPAI). For decades, avian influenza viruses were generally considered to be poorly pathogenic to humans [1].

The HPAI A(H_5_N_1_) influenza virus was first identified in 1996 in domestic waterfowl in Southern China, and named A/goose/Guangdong/1/1996. In 1997, poultry outbreaks were detected in China and Hong Kong and 18 human cases (including 6 fatalities) were identified. H_5_N_1_ re-emerged in 2003 leading to widespread outbreaks in poultry across Asia and a larger epidemic. The hemagglutinin (HA) gene diversified into many genetic groups (“clades”), and genetic lineages (“genotypes”) were identified across the Eastern Hemisphere: since 2005 wild bird migrations spread H_5_N_1_ to poultry in Africa, the Middle East and Europe (clade 2.2). Gene reassortment of H_5_ viruses from poultry and wild birds led to emergence of H_5_N_6_ and H_5_N_8_ virus subtypes, and HA diversified further into clade 2.3.4.4. Since 2005, the A(H_5_N_8_), and since 2013 the A(H_5_N_6_) influenza viruses have become the predominant HPAI subtypes detected globally, replacing most of the original H_5_N_1_ virus, resulting in sporadic confirmed human cases (7 for A(H_5_N_8_) and >70 for A(H_5_N_6_)). HA diversified further into clade 2.3.4.4b and became the predominant clade circulating in Asia, Africa, Europe, and the Middle East. Since 1997, 896 H_5_N_1_ cases (888 of which occurred since 2003) have been reported from 23 countries (most cases showing severe pneumonia, with >50% mortality), but very few cases were reported worldwide since 2015–2016: about half of the characterized viruses contain more than one mutation in the PB2 protein (E627K, D701N, or T271A) associated with increased virulence and/or replication in mammals, but no changes in the receptor binding due to associated HA mutations have been reported yet. Since 2016, HPAI A(H_5_N_1_) clade 2.3.4.4 has circulated and diversified so much in wild-birds [2] that in 2020 the WHO proposed eight genetic groups (named from a to h) [3].

## 2. The H_5_N_1_ Enzootics since 2022

The avian influenza A-H_5_N_1,_ H_5_N_2_, H_5_N_4_, H_5_N_5_, H_5_N_6_, and H_5_N_8_ subtypes have all been identified in poultry or wild birds. From December 2023 to February 2024, at least 646 HPAI outbreaks have been reported in five regions caused mainly by A(H_5_N_8_). Clade 2.3.4.4b A(H_5_N_1_) influenza viruses became the predominant subtypes in Asia, America, Africa, Europe, and the Middle East by the end of 2021, and were also detected in Antarctica in 2024 [4], representing a real enzootic. Co-infection of clade 2.3.4.4b H_5_N_1_ and H_5_N_6_ has been reported in ducks [5]. Since the beginning of 2021, massive circulation of clade 2.3.4.4b H_5_N_1_ in birds has led to tens of detections of A(H_5_N_1_) in humans: China (one in 2022 and one in 2023), Chile (one in 2023), Ecuador (one in 2022), Spain (two in 2022), the United Kingdom (one in December 2021 and four in April–June 2023), and the United States of America (USA) (one in April 2022 in Colorado, and one in Texas, two in Michigan, and ten in Colorado in 2024). No evidence of human-to-human transmission has been found yet.

Globally, in 2021–2023, several large outbreaks of clade 2.3.4.4b were reported in mammals from Italy [6] (farmed pigs), the Netherlands [7] (wild red foxes), Canada [8,9] (harbor and gray seals), the USA [10] (seals and dolphins [11]), Spain (farmed minks) [12], Chile [13], Argentina [14] and Peru [15,16] (sea lions and seals), Poland [17,18] (domestic cats), Finland [19] (farmed minks), South Korea [20] (cats), the USA [21] (dogs), and the UK [22] (dogs). Some viruses resulted in severe disease in infected ferrets [2,23]: transmission between ferrets by direct contact [24] and airborne [25] has been confirmed.

The virus from the human case in Chile had two substitutions (PB2-D701N and PB2-Q591K), both previously seen in A(H_1_N_1_)v. Since 2022 in Lao and Vietnam, and since 2023 in Cambodia, a novel reassortant influenza A(H_5_N_1_) virus was detected in poultry: it caused the human cases reported from Cambodia since late 2023 and from Vietnam in March 2024, all harboring PB2:E627K. Such lineage contains the surface proteins from clade 2.3.2.1c that has circulated locally, but internal genes are from a more recent clade 2.3.4.4b virus.

While clade 2.3.4.4b was not among the predicted top emerging influenza viruses according to the CDC Influenza Risk Assessment Tool (IRAT) [26], since Spring 2024 the USA are experiencing an unprecedented outbreak (affecting 178 ranches across 13 states at the time of writing) in lactating Holstein cows, due a single introduction of genotype B3.13 from birds into cattle [27,28]: this genotype contains PA, HA, NA, and M gene segments from Eurasian wild bird lineages and PB2, PB1, NP, and NS gene segments from North American 2.3.4.4b wild bird lineages. The movement of asymptomatic cattle has likely played a role in the spread of HPAI within the USA dairy herd [28], and reverse transmission from cows to birds has been documented [29,30]. In 2008, cattle had been previously experimentally shown to be a replication-competent host for a different H_5_N_1_ virus (A/cat/Germany/R606/2006) [31].

The first set of viral sequences from the US outbreak was released on 21 April 2024 by the USDA/APHIS in the Sequence Read Archive (SRA) of the National Institute of Health’s National Library of Medicine, National Center for Biotechnology Information (NCBI), and genome consensus was assembled by a team of scientists on April 29 [32]: such posted sequences were soon used to construct phylogenetic trees [33].

All eight HPAI A(H_5_N_1_) isolates derived from dairy cattle and two cats in Texas in March 2024 demonstrated the presence of HA:137A, 158N, and 160A mutations [34] (which may increase the binding affinity to the human-type receptor [35,36]), while none contained the residues 192I, 225D, or 228S [34]: without such mutations H_5_N_1_ attached only rarely to epithelial cells or goblet cells, and only occasionally to cells of the submucosal glands [37]. Furthermore, all isolates exhibited the residues 30D, 43M, and 215A in M1 [38,39,40], as well as 42S, 103F, and 106M in NS1 [40]. All of the detected HA and M mutations pre-existed in circulating avian lineages [34]. It is noteworthy that the mutations Q591K, E627K/V/A, or D701N in PB2 (previously associated with mammalian host adaptation and enhanced transmission [39,41,42]) were absent in all eight isolates [34], but all had PB2:M631L, an alternative putative human adaptive mutation. Twelve viral sequences from cow milk from Minnesota exhibited PB2:Q591R, a mutation associated with mammalian adaptation in H_1_N_1_v in the absence of PB2:E627K [43]. Overall, B3.13 is a reassortant of six different sublineages (H_5_N_1_, two different H_11_N_9_, two different H_3_N_8_, and H_6_N_2_) [34].

Several cattle sequences from Kansas exhibited both PB2:R340K (a mutation known to increase pathogenicity and transmissibility in mice and guinea pigs in H_9_N_2_, even more when co-occurring with PB2:588V [44]) and PB2:E249G (a mutation known to increase mammalian adaptation in H_3_N_2_ [45]): intriguingly, the sequence from Colorado contains the unique PB2:E249G and NS1:R21Q found in environmental samples in Kansas [46].

As expected from historical evidence supporting cows as susceptible to influenza virus D and permissive but resilient to infection with influenza viruses A, B, and C, A(H_5_N_1_)-infected cattle experienced only self-limiting reduced feed intake and rumination and an abrupt drop in milk production. On the contrary, a fatal systemic infection (including meningoencephalitis and myocarditis) developed in domestic cats fed raw (unpasteurized) colostrum and milk from A(H_5_N_1_)_-_affected cows [47]. At least 11 house mice also tested positive near an affected dairy farm in Roosevelt, New Mexico, at the beginning of May 2024. The latter finding was not unexpected, given knowledge from tissue distribution of SA receptors in cats [48], cat challenge studies [49], necropsies from free-living mesocarnivores [8], and recent outbreaks in cats and in Poland [17,18] and South Korea [20]. The B3.13 outbreak has also involved an alpaca.

Lectin binding studies showed a lack of the receptors considered necessary for efficient respiratory transmission of avian influenza viruses [50] and, accordingly, calves inoculated experimentally with H_5_N_1_ were poorly susceptible and did not transmit virus to contact animals [31]. Nevertheless, experiments dating back to 1953 confirmed that the bovine mammary gland was competent for influenza virus propagation [51], and the same applied to ferret mammary gland, as shown in 2015 [52]. Earlier research also showed that getting virus-filled milk in the eye area could lead to an eye infection.

Since viable and infectious virus has been recovered in unpasteurized milk on milking machinery surfaces [53,54], cow-to-cow transmission has been suspected to happen via contaminated teat cups of milking machines [55] (H_5_N_1_ replicating in epithelial cells of mammary alveoli [47]) causing mastitis (and being excreted in milk), but could as well be wind-borne [56] or transmitted via mechanic vectors such as house flies [57]: aerosol challenge and sentinel studies in both lactating and nonlactating cows [58] have shown mild symptoms in heifers [59]. Additionally, aerosols could be generated when using high-pressure hoses in the milking parlor to clean the floors of feces, urine, and spilled milk. The duck (SA-α2,3-Gal-β1,3) and human (sialic acid (SA)-α2,6) influenza A virus receptors (but not the chicken receptor SA-α2,3-Gal-β1,4) were widely expressed in the bovine mammary alveoli (but not ducts), whereas the chicken receptor dominated in the respiratory tract, and only a low expression of receptors was observed in the neurons of the cerebrum [60]. Nevertheless, the SNA lectin the authors used to identify SA-α2,6 cannot distinguish between Neu5Ac and the (non-human) Neu5Gc form of the a2,6-linkage that is abundant in bovines [61,62,63]. Another group found that the B3.13 H_5_ protein binds well in the mammary gland (the only tissue with the 5-*N*-glycolyl modification required for HA binding), whereas the classical H_5_ proteins do not [64]. B3.13 (but also an older HPAI H_5_N_1_ isolate) spread systemically to mice and ferrets, including their mammary glands, but only the B3.13 virus bound to sialic acids expressed in human upper respiratory airways and inefficiently transmitted to exposed ferrets (only one out of four exposed ferrets seroconverted without virus detection) [65].

On 25 April 2024, 58 out of 150 (39%) of commercial milk products from around the Midwestern USA tested by academics turned out to be positive for H_5_N_1_ viral RNA, an estimate later tapered at 20% by the FDA. Although the risk of transmission from pasteurized milk remains low [66,67,68,69,70] (with 297 egg inoculation tests being negative as of 1 May 2024 according to the FDA), the very high viral loads detected in fresh milk pose a concern for environmental contamination. Thermal inactivation and replication in bovine kidney and lung cells of clade 2.3.4.4b A(H_5_N_1_) in milk is strain-dependent and not affected by fat content [71]. Dairy farms are not usually plumbed into municipal wastewaters and do not have their own septic systems to handle large amounts of wasted milk: when left in manure pits or lagoons, infected raw milk can propagate the epizootics. Surveys of retail milk in Canada were negative so far [72,73].

Other polymerase mutations of potential interest present to some degree in the cattle sequences are PA:497 and PA:613. The NA of genotype B3.13 has a full-length stalk and no mutations linked to resistance to neuraminidase inhibitors (NAI); nevertheless, one case had the mutation NA:T438I that confers resistance to NAIs (as for the NA:S246N recently found in a dolphin in Florida [11]), and the use of generic oseltamivir for veterinary purposes remains uninvestigated.

The viral sequence from the human case from Texas, who had had professional exposure and fully recovered after experiencing conjunctivitis [74], harbored PB:E627K (as found in a single cow in Texas [28]) but not M631L (found in all cattle sequences) [74], suggesting the human was infected by a lineage still undetected in cattle. A second case in a dairy worker with conjunctivitis was confirmed in Michigan on 22 May, which harbored PB2:M631L but not E627K. A third case in a dairy worker with respiratory symptoms from Michigan was reported on 30 May. A fourth case in a dairy worker with conjunctivitis as reported from Colorado on 3 July, and nine more were reported from Colorado among poultry cullers at two different farms the end of July 2024. The latest development is of high concern, since it suggests that a new reservoir of partly mammalian-adapted H_5_N_1_ has been created in US cattle that can keep spilling over into poultry (and unprotected poultry-exposed humans) all year long. In summary, PB2:M631L represents the signature mutation for the dairy cattle outbreak: the main mutations seen in human cases in HA are N110S, L131Q, T139P, and V226A, while the main mutations in NA are instead H155Y and T188I.

Notably, only 23 people across eight states had been tested for H_5_N_1_ as of 25 April, which increased to 300 as of 19 May, making it likely that more cases have remained undetected: it should be noted that 50% of dairy farm workers are undocumented immigrants with language barriers and a lack of trust in government institutions. Furthermore, 30 April marked the end of the COVID-era mandate to report hospital admissions and capacity and occupancy data to the federal government, hindering epidemiological surveys, but monitoring conjunctivitis incidence could prove extremely useful [63]. Seroprevalence in cattle has not been systematically investigated since the outbreak, which represents another major fault. The prevalence of H_5_N_1_-neturalizing antibodies among cattle workers was first reported at 14.3% (out of just 14 workers at two farms) at the end of July 2024 [75], but low titers suggest that the microneutralization assay used could have detected cross-reactive anti-stalk antibodies from seasonal H_1_N_1_. Wastewater-based epidemiology has been successful during the COVID-19 pandemic [76], and can be exploited again to monitor A(H_5_N_1_): out of 159 wastewater treatment plants that currently monitor sludge samples for influenza A, 59 saw increases in the A-strain viruses in Spring 2024, while numbers of human flu cases were consistent or falling: wastewaters in Texas around affected ranches have shown peaks of H_5_N_1_, especially where industrial discharges containing animal waste, including milk byproducts, were permitted to be discharged into sewers [77]. In a related study, clade 2.3.4.4b was detected in 19 out of 23 monitored wastewater sites across nine cities in Texas [78]. Regardless of this, the CDC is currently not recommending wastewater monitoring for A(H_5_N_1_) given the troubles at imputing its origin. However, even if it would prove entirely from cows or avians, the data would anyway be informative, so we disagree with this decision.

Testing of environmental samples at an affected dairy farm revealed two newly emerged mutations in the PB2 (E249G) and NS1 (R21Q) genes, but the mammalian adaptation mutation PB2:E627K was found in only 1.7% of the sequence reads. Such environmental samples had PB2 and NS similar to the human case from Texas, while HA and NA were identical to those from affected dairy cattle [46].

## 3. H_5_N_1_ Vaccines for Human Use

While prevention remains the best option, e.g., by avoiding ruminants to be fed over poultry litters, pandemic preparedness requires an assessment of available or upcoming antiviral therapeutics and vaccines. We recently reviewed therapeutics for influenza viruses and the many uncertainties that still remain about their efficacy [79]: the PENINSULA RCT of pre-exposure prophylaxis with the anti-HA monoclonal antibody (mAb) VIR-2482 recently failed [80].

In this manuscript, we will summarize the authorized anti-H_5_ vaccines (Table 1) and the clinical pipeline. Pandemic preparedness is partly based on the stockpiling of pre-pandemic candidate vaccine viruses (CVVs): despite the fact that CVVs will likely have a partial match with the pandemic strain, immunologic priming [81] will likely help first responders and patients at risk of complications to buy time before a better-matched vaccine is manufactured and distributed.

In the United States, the National Prepandemic Influenza Vaccine Stockpile (NPIVS) [82] currently includes four H_5_N_1_ avian influenza strain vaccines (clade 1, A/Vietnam/1203/2004; clade 2.1.3, A/Indonesia/05/2005; clade 2.2, A/bar-headed goose/Qinghai/1A/2005; and clade 2.3.4, A/Anhui/1/2005). The NPVIS also includes two proprietary squalene-based adjuvants (GSK’s AS03 and Novartis’ MF59), since typically pandemic strains have poor immunogenicity in unprimed populations [83,84]. AS03 comes with several concerns, since in 2012–2013 several European nations reported an association between narcolepsy and an A(H_1_N_1_)pdm09 vaccine that used AS03 [85], at a rate of 1 case every 16,000–50,000 doses [86]. Antibodies to influenza nucleoprotein (NP, a minor antigen in split-virion vaccines) cross-react with human hypocretin receptor 2 (HCRTR2) [87,88] in genetically susceptible individuals, and Pandemrix^®^ was found to include a higher amount of denatured nucleoproteins than other pandemic vaccine brands [89]. Notably, GSK has since moved to the AS04 adjuvant, which consists of aluminum hydroxide and monophosphoryl lipid A (MPL).

In the USA, there are two adjuvanted, inactivated monovalent H_5_ vaccines that are FDA-approved for those aged 6 months or older and which have to be administered as two doses 21 days apart, but none of them is commercially available at the time of writing [90].

- On 14 November 2013, the FDA licensed the ID Biomedical Corporation’s (a Canadian subsidiary of Glaxo Smith Kline Biologicals) egg-based Influenza A (H_5_N_1_) Virus Monovalent Vaccine, including A/Indonesia/05/2005 (H_5_N_1_) and the AS03 adjuvant [91], but the vaccine was not commercially available: the US federal government purchased it for the NPIVS for as-needed distribution [92].
- On 31 January 2020, the US Food and Drug Administration (FDA) authorized the use of CSL Seqirus Inc.’s Audenz™ (Holly Springs, NC, USA) subunit influenza vaccine prepared from A/turkey/Turkey/1/2005 NIBRG-23 virus propagated in Madin Darby Canine Kidney (MDCK) cells, adjuvanted with MF59. The vaccine was manufactured by I.D. Biomedical Corporation.

According to the US’ CDC, about 20 million H_5_N_1_ and 12 million H_7_N_9_ vaccine doses were available in the NPIVS as of June 2023, as were two H_5_ CVVs similar to the HA protein of H_5_N_1_ clade 2.3.4.4b A(H_5_) CVV. Indeed, the HA of genotype B3.13 involved in the 2024 US cattle outbreak is very closely related to the A/Astrakhan/3212/2020-like CVV (IDCDC-RG71A) (just differing for L104M, L115Q, T195I, V210A) and the A/American wigeon/South Carolina/22-000345-001/2021-like CVV (IDCDC-RG78A) (just differing for L104M and L115Q). The US Biomedical Advanced Research and Development Authority (BARDA) has agreements in place with Vir Biotechnology and CSL Seqirus.

The Japanese National Institute of Infectious Disease (NIID) is developing a CVV based on A/Ezo red fox/Hokkaido/1/2022-like 2.

The European Medicines Agency (EMA) has so far evaluated several products:

- Aflunov™, which contains an A/turkey/Turkey/1/2005 (H_5_N_1_)-like strain (NIBRG-23) (clade 2.2.1) was submitted but the application was withdrawn on June 2008.
- Celldemic™ and Incellipan™ (zoonotic influenza vaccine (H_5_N_1_) (surface antigens, inactivated, adjuvanted, prepared in cell cultures) were authorized in April 2024.

**Table 1:** Summary of inactivated, intramuscular pandemic influenza vaccines authorized for human use. Vaccines against pandemic H_1_N_1_pdm09 are reported for historical comparison.

Target Influenza	Brand Name	Manufacturer	Used Lineage	Cell Substrate	Antigen	Adjuvant	Regulatory Status	Availability
Pandemic H_1_N_1_pdm09	Pandemrix™	GlaxoSmithKline Biologicals S.A. (Rixensart, Belgium)	A/California/7/2009 (H_1_N_1_)v-like strain X-179A (derived from A/Puerto Rico/8/1934)	11-day-old fertilized hens’ eggs	Split virion	AS03	Administered outside USA	EMA marketing authorization expired August 2015
	Arepanrix™							Unknown
	Focetria™	Novartis Vaccines and Diagnostics S.r.l. (Siena, Italy)	A/California/7/2009 with HA1:N146D		Surface	MF59C.1		Unknown
	Celvapan™	Nanotherapeutics Bohumil, s.r.o. (Jevany, Czech Republic)	A/California/7/2009	Vero cells	HA	AS03		Unknown
	Panvax™	CSL Biotherapies Ltd. (Melbourne, Australia)	A/California/7/2009 (H_1_N_1_)v-like	11-day-old fertilized hens’ eggs	Split-virion	no	Approved by TGA (Australia), FDA, EMA, Singapore, Germany, and New Zealand [93]	Unknown
Prepandemic H_5_N_1_	none	ID Biomedical Corp (Vancouver, Canada), acquired by GSK in 2005	A/Indonesia/05/2005 (H_5_N_1_) (clade 2)	11-day-old fertilized hens’ eggs	Split-virion	AS03	Approved by FDA	US stockpile only
	Influenza Virus Vaccine, H_5_N_1_	Sanofi Pasteur (Lyon, France)	A/Vietnam/1203/2004 (H_5_N_1_, clade 1)		Split-virion	No	Approved by FDA since 2007 for national stockpile	Unknown
	Audenz™	Seqirus Inc, USA, now part of CSL Seqirus (Melborne, Australia)	A/turkey/Turkey/1/2005 (H_5_N_1_)-like strain (NIBRG-23) (clade 2.2.1)	MDCK cells	Subunit	MF59	Approved by FDA	Unknown
	Incellipan™	Seqirus Netherlands B.V. (Amsterdam, The Netherlands)			HA and NA	MF59C.1	Authorized by EMA	Unavailable
	Celldemic™						Authorized by EMA	Unknown
	Aflunov™	Novartis Vaccines and Diagnostics S.r.l. (Siena, Italy)		11-day-old fertilized hens’ eggs			EMA request withdrawn in 2008	Unknown
	Foclivia™	Seqirus S.r.l. (Siena, Italy)	A/Vietnam/1194/2004 (H_5_N_1_) (clade 1)				Authorized by EMA	Unknown
	Pumarix™	GSK Biologicals S.A. (Rixensart, Belgium)	A/Indonesia/05/2005 (H_5_N_1_) like strain used (PR8-IBCDC-RG2) (clade 2)		Split virion	AS03	Authorization withdrawn by EMA	Unavailable
	Prepandrix™ (formerly GSK1557484A [94,95])						Authorization withdrawn by EMA	Unavailable
	Adjupanrix™ (formerly GSK1562902A) [96,97,98,99]		A/VietNam/1194/2004 NIBRG 14 (clade 1)				Authorized by EMA	Unknown
	Panflu™	Sinovac Biotech Ltd. (Beijing, China)	A/Vietnam/1194/2004-A/PR/8/34 (NIBRG-14)		Whole-virion	Yes	Approved in China	Unknown

The European Commission (EC) signed a framework contract on 28 July 2022, for the possible joint procurement of 85 million vaccine doses of GSK’s adjuvanted, inactivated split virion H_5_N_1_ vaccine Adjupanrix™ based on A/VietNam/1194/2004 NIBRG 14 [100].

Other countries are developing stockpiles. In May 2023, the Japan Health Ministry announced that it is changing its 10 million influenza vaccine stockpile from H_7_N_9_ to the H_5_N_1_ virus. On 26 September 2023, the UK’s Health Security Agency announced, an advance purchase agreement with CSL Seqirus to produce over 100 million influenza pandemic vaccines if or when they are needed.

Many more CVVs have been successfully manufactured by other companies but are not within national stockpiles, e.g., Sanofi Pasteur manufactured an investigational subvirion inactivated monovalent influenza vaccine (IIV) from the A/Indonesia/05/2005 (H_5_N_1_) PR8-IBCDC-RG2 influenza virus, clade 2.1.3, adjuvanted with AS03 [101].

While we have CVVs, the main hurdle in case of a pandemic remains scaling-up of manufacturing. The current flu vaccine manufacturing capacity (including both egg-based and cell-based manufacture) is about 1.2 billion doses of trivalent vaccines, which corresponds to 3.6 billion doses of 15 µg monovalent (pandemic) vaccines, i.e., 1.8 million of 15 µg 2-dose courses. >85% of this manufacturing capacity stays with seven producers (CLS Seqirus, Novartis, GSK), and only 2% stays in low- and middle-income countries (LMICs) (which make up 38% of the world’s population). This clearly generates an equity issue, since >95% of the doses manufactured each year are used in high- and upper/middle-income countries. Assuming that for a less immunogenic avian influenza vaccine we need a higher antigen level per dose (i.e., 90 µg), there will be capacity for only 0.3 million 2-dose courses. In case we can spare antigens (down to 7.5 µg per dose) by using adjuvants, this estimate could be increased to 3.6 million 2-dose courses.

## 4. Production Technologies

**Figure 1:**
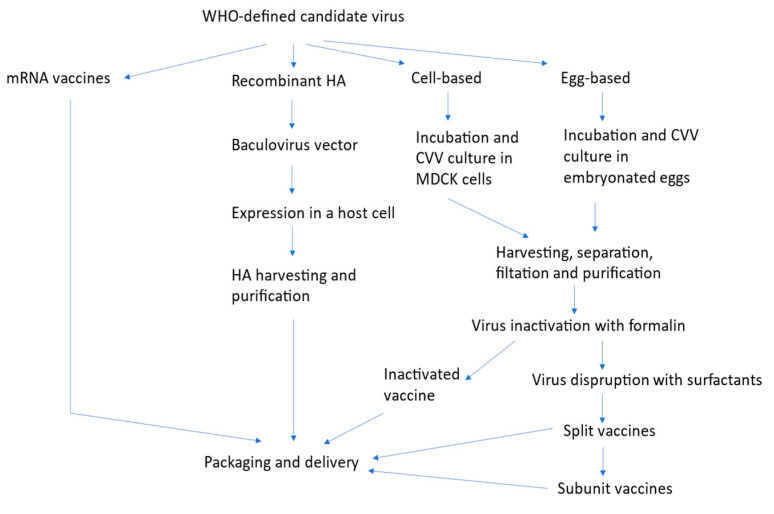
Influenza vaccine manufacturing processes.

Several influenza vaccine manufacturing processes are possible nowadays (Figure 1) [102,103]. Under the currently most commonly used production technology, hundreds of millions of 11-day-old fertilized hens’ eggs are needed to produce the required amount of protein antigens, and the world is currently suffering an egg shortage [104]. While such system cannot meet surge capacity requirements, it is even more challenged by the scenario of bird flu, which could ravage hens. Although flocks of hens used for egg production are housed in biosecurity facilities and are segregated from the food supply, they nevertheless require periodic replacement. Recently, Madin−Darby canine kidney (MDCK) cells have been used to replace eggs and avoid egg adaptation of the virus.

Historically, live attenuated influenza vaccines (LAIV) were first proposed in the 1960s: cold-adapted donor viruses are passaged with a gradual reduction in the temperature in embryonated chicken eggs. The final lineage can only replicate at 20 °C, i.e., only within the nasopharynx where they are usually administered, inducing mucosal immunity.

Alternatively, virions are chemically inactivated, mainly with formaldehyde or β-propiolactone, then purified intact (whole-virion inactivated vaccines, WIV) or split by disrupting the membrane with the nonionic surfactants Triton^®^ X-100 or octyl glycoside (split-virus inactivated vaccines, SIV). Surfactants can be further used to split the virion into subunits (subunit vaccines). Today, individual subunits can be manufactured with recombinant protein technology via baculovirus vectors (recombinant vaccines) [103]. While recombinant vaccines are less reactogenic and devoid of mutations, they are also less immunogenic and require dose escalation.

Changing delivery from intramuscular to intradermal or adding adjuvants could spare antigens, and this approach has been advocated for decades [105].

Novel vaccine platforms are under development to match cost with scalability and strain stability: examples include virus-like particles (VLP), antigen-presenting cell (APC)-inducible vaccines, viral vector vaccines, nanoparticle-based influenza vaccines, and nucleic acid vaccines (DNA or mRNA).

## 5. Lessons Learnt from H_5_N_1_ Clinical Trials

Influenza A vaccine efficacy has historically been quite low and variable on a year-by-year basis according to how good the match has been for that specific year (the vaccine being designed according to predictions attempted months before the epidemic season): efficacy ranges from 28 to 64% in those aged 18–64 years and is even lower (17–57%) for those aged >65 years. Reactogenicity has generally been lower with subunit vaccines or SIV than with WIV, while immunogenicity has generally been higher for WIV than for SIV. The following paragraphs will focus on experiences with A(H_5_N_1_) vaccines.

At the time of writing, PubMed reports 194 clinical trials on vaccines against H5N1, the last one from 2021, ranging from Matrix-M-adjuvanted VLP [106] to AS03- [107] or MF59- [108] adjuvanted cell-derived vaccines, to adenovirus-vectored vaccines [109], to replication-deficient vaccines [110]. Overall, immunogenicity and safety have been very high for all the formulations, leaving all opportunities open for further development.

Heterologous boosting seems an effective strategy to increase immunogenicity. Previous priming with live attenuated influenza A(H_5_N_1_) vaccines (pLAIVs) followed by one dose of an inactivated subvirion influenza A(H_5_N_1_) vaccine leads to higher antibody titers, affinity, and breadth than delivering two doses of an inactivated subvirion influenza A(H_5_N_1_) vaccine [111].

High titers of cross-reactive NA-inhibiting antibodies to clade 2.3.4.4b were detected in 97% of serum samples from healthy adults in Hong Kong in 2020, while low titers were detected in 42% of serum samples collected in 2009, before A(H_1_N_1_)pdm09 exposure. Influenza A(H_1_N_1_)pdm09 and A(H_5_N_1_) titers were correlated, suggesting some serological cross-reactivity between the two lineages [112].

mRNA vaccines have the potential to combine multiple HA antigens at tapered costs, paving the way to universal influenza A and B vaccines (with only one lineage of the latter still circulating at the time of writing [113]), such as the one reported in November 2022 by the University of Pennsylvania to protect mice and ferrets [114]. The Duke Human Vaccine Institute, a part of NIAID’s CIVICs network, is similarly testing a trial (NCT05755620) of the mRNA equivalent of an influenza H1 hemagglutinin-stabilized stem ferritin nanoparticle vaccine [115,116]. CSL has phase I trial (NCT06028347) based on self-amplifying messenger RNA (sa-mRNA) [117] influenza vaccine candidates [118], and mRNA vaccines against A(H_5_) are also under phase I clinical trials by GSK (NCT06382311) and ModernaTx (NCT05972174) after preliminary success against H_10_N_8_ and H_7_N_9_ [119].

Experiences from the late COVID-19 pandemic have clearly established that COVID-19 and influenza vaccines can be safely co-administered without compromising their efficacy [120]. Nevertheless, a few concerns remain regarding side effects in selected frail populations: historically, influenza vaccination (as most other vaccines) has been associated with graft rejection [121] and lymph node hyperplasia simulating relapses of lymphoma [122].

## 6. Conclusions

With only a few tens of human H_5_N_1_ cases confirmed since 2021, and no evidence for human-to-human transmission, we are still in time to slowly prepare for this virus. Despite decades of good research on influenza viruses, we are still getting surprises from nature, e.g., the discovery of avian and human influenza virus receptors in bovine mammary alveoli. While historically, pigs, harboring a breadth of HA subtypes [123], have been considered the mixing vessel for novel influenza pandemics, similar concerns exist for cats [124] and dogs [125], and cows are also on the list [126]. We have been used to thinking that the next influenza pandemic will emerge in Southeast Asia, like many times in the past, where wild birds, poultry, pigs, and people mix in the marketplaces. Nevertheless, intense farming practices create opportunities for the virus to evolve in the Western world.

Pigs are highly susceptible to, but do not transmit, mink-derived clade 2.3.4.4b [127]. Nevertheless, for these species to act as efficient mixing vessels, co-expression of avian and human receptors in the upper respiratory tract is needed, as well as hyperendemicity of species-specific influenza viruses: this has occurred in pigs but not yet in other species. Although in the ongoing US outbreak cow-to-cow transmission has been proven to be largely driven by contaminated mechanical vehicles, and its interstate spread to be traffic-driven (and hence easier to contain), the risk of environmental spilling should not be minimized.

Recombination between non-2.3.4.4b LPAI and 2.3.4.4b HPAI remains a totally unpredictable event, as happened in a 59-year-old male fatally affected by H_5_N_2_ in Mexico City in April 2024 [128].

The influenza virus polymerase consists of the polymerase acidic (PA), polymerase basic 1 (PB1), and polymerase basic 2 (PB2) subunits: it transcribes viral genes and replicates the vRNA in the nucleus of infected cells. While PB2 and PA exploitation of host acidic nuclear phosphoprotein 32 (ANP32A and ANP32B) remains the most investigated target for mammalian adaptations [129,130], more mutations are needed at different genes for a successful adaptation to humans. For example, ANP32E can be exploited in H_9_N_2_ by PB1:K577E [131], which was found in Texas cattle in May 2024. Deep mutation scanning has recently been performed for both H5 hemagglutinin [132] and nuclear export protein (NEP) [133]. No study to date has assessed the trend for mutations known to evade human BTN3A3, a recently discovered interferon-stimulated gene (ISG) that selectively restricts infections from avian influenza viruses [134] in all primates by recognizing the viral nucleoprotein (NP). Evasive mutations at a 3D site have been defined as NP:Y52H/V/Q and NP:F313L/S/V [135]. Notably, all of the 20th century pandemic influenza A viruses were BTN3A3-resistant thanks to either F313Y (1918, H_1_N_1_; 1957, H_2_N_2_; and 1968, H_3_N_2_) or F313V (2009, H_1_N_1_), and Y52N acquired in avian sublineages H_7_N_9_ [135] and H_5_N_1_ [136] likely facilitated human infections.

When it comes to pandemic preparedness, many faults still persist, e.g., commercial at-home and point-of-care tests are currently unable to discriminate HA serotypes, passive immunotherapies require administration very early after symptoms develop, and convalescent plasma (the easier-to-scale front-line treatment) is still missing a regulatory status. Anti-influenza therapeutic mAbs will hardly be economically sustainable to low- and middle-income countries, and scalability of manufacturing remains challenging. Progress has been made in vaccine manufacturing, with a pipeline richer than ever before, but the mRNA and viral vector platforms remain unlicensed for influenza vaccines. The WHO’s Global Influenza Surveillance and Response System (GISRS) has identified two CVVs that should match the current B3.13 genotype, but it is reasonable to assume that the final pandemic clade will somewhat differ from the current ones because of ongoing evolution. As such, existing stockpiles could be left minimally effective and in low amount because of their progressive expiry.
